# Outcome preferences of older people with multiple chronic conditions and hypertension: a cross-sectional survey using best-worst scaling

**DOI:** 10.1186/s12955-019-1250-6

**Published:** 2019-12-19

**Authors:** Hélène E. Aschmann, Milo A. Puhan, Craig W. Robbins, Elizabeth A. Bayliss, Wiley V. Chan, Richard A. Mularski, Renée F. Wilson, Wendy L. Bennett, Orla C. Sheehan, Tsung Yu, Henock G. Yebyo, Bruce Leff, Heather Tabano, Karen Armacost, Carol Glover, Katie Maslow, Suzanne Mintz, Cynthia M. Boyd

**Affiliations:** 1 Epidemiology, Biostatistics and Prevention Institute, University of Zurich, Zurich, Switzerland; 2 Center for Clinical Information Services, Kaiser Permanente Care Management Institute, Oakland, CA USA; 3 Kaiser Permanente National Guideline Program, Oakland, CA USA; 4 Guidelines International Network, Board of Trustees, Denver, CO USA; 5 Family Medicine, Colorado Permanente Medical Group, Denver, CO USA; 6 Clinical Education MOC Portfolio, The Permanente Federation, Oakland, CA USA; 7 Institute for Health Research, Kaiser Permanente, Denver, CO USA; 8 Department of Family Medicine, University of Colorado School of Medicine, Aurora, CO USA; 9 Kaiser Permanente Northwest, National Guideline Program, Portland, OR USA; 10 The Center for Health Research, Kaiser Permanente Northwest, Portland, OR USA; 11 Department of Pulmonary & Critical Care Medicine, Northwest Permanente, Portland, OR USA; 12 Oregon Health & Science University, Portland, OR USA; 13 Department of Health Policy and Management, Johns Hopkins University Bloomberg School of Public Health, Baltimore, MD USA; 14 Division of General Internal Medicine, Johns Hopkins University, School of Medicine, Baltimore, MD USA; 15 Division of Geriatrics and Gerontology, Johns Hopkins University, School of Medicine, Baltimore, MD USA; 16 Department of Public Health College of Medicine, National Cheng Kung University, Tainan, Taiwan; 17 Division of Geriatrics and Gerontology, Patient and Caregiver Partner Group, Johns Hopkins University School of Medicine, Baltimore, MD USA; 18 Gerontological Society of America, Washington, District of Columbia, USA; 19 Family Caregiver Advocacy, Kensington, MD USA

**Keywords:** Patient preferences, Multiple chronic conditions, Hypertension, Outcome preferences, Best-worst scaling

## Abstract

**Background:**

Older people with hypertension and multiple chronic conditions (MCC) receive complex treatments and face challenging trade-offs. Patients’ preferences for different health outcomes can impact multiple treatment decisions. Since evidence about outcome preferences is especially scarce among people with MCC our aim was to elicit preferences of people with MCC for outcomes related to hypertension, and to determine how these outcomes should be weighed when benefits and harms are assessed for patient-centered clinical practice guidelines and health economic assessments.

**Methods:**

We sent a best-worst scaling preference survey to a random sample identified from a primary care network of Kaiser Permanente (Colorado, USA). The sample included individuals age 60 or greater with hypertension and at least two other chronic conditions. We assessed average ranking of patient-important outcomes using conditional logit regression (stroke, heart attack, heart failure, dialysis, cognitive impairment, chronic kidney disease, acute kidney injury, fainting, injurious falls, low blood pressure with dizziness, treatment burden) and studied variation across individuals.

**Results:**

Of 450 invited participants, 217 (48%) completed the survey, and we excluded 10 respondents who had more than two missing choices, resulting in a final sample of 207 respondents. Participants ranked stroke as the most worrisome outcome and treatment burden as the least worrisome outcome (conditional logit parameters: 3.19 (standard error 0.09) for stroke, 0 for treatment burden). None of the outcomes were always chosen as the most or least worrisome by more than 25% of respondents, indicating that all outcomes were somewhat worrisome to respondents. Predefined subgroup analyses according to age, self-reported life-expectancy, degree of comorbidity, number of medications and antihypertensive treatment did not reveal meaningful differences.

**Conclusions:**

Although some outcomes were more worrisome to patients than others, our results indicate that none of the outcomes should be disregarded for clinical practice guidelines and health economic assessments.

## Background

In older people with multiple chronic conditions (MCC) treatment is often complex and burdensome [1]. When considering prevention of cardiovascular disease in older people with MCC and hypertension, there is a trade-off between prioritizing treatments to achieve long-term goals, and avoiding treatment burden and side effects. This trade-off usually depends on the individual’s health profile and preferences.

A previous study involving patients and caregivers identified the question of how intensively to lower blood pressure in people with MCC as a top priority question to answer [2]. However, empiric evidence on preferences of people with MCC for patient-important outcomes related to hypertension to inform this question is lacking. This evidence is crucial, because how a patient values different health outcomes related to hypertension will determine the trade-off of whether to start or intensify antihypertensive treatment [3], and also related questions such as which medication to add. Evidence about patient preferences is essential to inform population-level decisions, such as clinical practice guidelines and health economic assessments, in a patient-centered manner [4]. For example, defining the relative importance of outcomes is critical in the development of clinical practice guidelines [5–7], and weighing outcomes differently (relative to one another) can shift the benefit-harm balance of an intervention [8]. Patient preferences can be considered quantitatively in guideline development to weigh benefits against harms [9].

While some studies have elicited patients’ preferences on benefits and harms related to hypertension treatment, they considered only a few of the possible outcomes or they combined outcomes, and did not recruit or report on people with MCC [10–13].

Therefore, our primary aim was to utilize best-worst scaling to elicit preferences about patient-important outcomes related to hypertension in people with MCC, in order to determine the relative importance that should be attributed to these outcomes in guideline development or policy-making. Our second aim was to explore whether preferences were associated with baseline characteristics.

## Methods

### Study design and setting

We conducted a cross-sectional survey to elicit preferences for outcomes related to the treatment of hypertension in individuals with MCC and hypertension. Participants were members of Kaiser Permanente Colorado, a not-for-profit integrated delivery system. Both the Institutional Review Boards of Johns Hopkins University and Kaiser Permanente Colorado approved this study.

### Eligibility

Using clinical and administrative data derived from the electronic health record and membership enrollment files, we identified individuals who were 60 years of age or older, had a history of hypertension, had one or more non-cardiovascular comorbidities, and had a score of 3 or more based on the Quan adaptation of the Elixhauser comorbidity index (Quan score) [14]. Non-cardiovascular comorbidities we considered were HIV/AIDS, alcohol abuse, anemia, chronic pulmonary disease, depression, dementia, drug abuse, liver disease, neurological disorders and other paralyses, cirrhosis, osteoarthritis, osteoporosis, peptic ulcer disease, psychoses, pulmonary/circulation disorders, renal failure and rheumatoid arthritis.

We excluded patients who were not fluent in spoken English and patients who were visually impaired (e.g., legal blindness). We included patients who were mildly cognitively impaired, but excluded people who had a diagnosis of dementia within the 365 days prior to the cohort creation.

### Sample recruitment

A random sample of eligible individuals was identified administratively using the Kaiser Permanente Colorado Virtual Data Warehouse, a quality-controlled common data model derived from multiple Kaiser Permanente Colorado data sources [15]. We recruited random samples of eligible participants in waves of 50 until we reached the target of 200 completed surveys. Potential participants received a recruitment mailing that included an invitation letter, a Study Information Sheet, an opt-out postcard, the paper survey with a postage paid return envelope, and a $10 gift card incentive. Potential participants received follow-up telephone calls after 2 to 4 weeks which served as reminders and also offers of assistance with survey completion if needed.

There is no sample size calculation for best-worst scaling [16, 17]. In a review of best-worst scaling surveys in health care [17], the median sample size among object case surveys was 180. We defined a target sample size of 200.

### Development of the best-worst scaling survey

We designed the survey as best-worst scaling tasks (case 1), a method introduced by Finn and Louviere [18]. In this design, respondents are asked to choose the best and the worst of three or more “objects”. The main advantage of this method is that it has more discrimination than for example discrete choice experiments, as it also elicits which is the worst object, and not only which is the best. Thereby, it can yield complete rather than partial ranking information [17]. Best-worst scaling is assumed to decrease cognitive burden placed on respondents, by asking to compare only a few of the outcomes at a time, instead of comparing all at once. We chose this method to minimize the cognitive burden, since we also included respondents with mild cognitive impairment, and because it allowed us to compare many outcomes. We used the balanced incomplete block design (generated using SAS version 9.4); the survey consisted of 11 blocks of five outcomes in total. As all outcomes had a negative impact on health, we phrased the question as: “If one of the following health problems were to happen to you, which would worry you most and which would worry you least?” The survey is shown in Additional file 1.

Based on previous input from patient and caregiver focus groups [2] and a literature review of outcomes that have been used in relevant clinical trials, we identified 12 patient-important outcomes (death, myocardial infarction, stroke, chronic heart failure, end-stage renal disease (with dialysis), chronic kidney disease, acute kidney injury, hypotension with dizziness, syncope, cognitive impairment, injurious falls and treatment burden). We included all except death in the survey. Based on another study [19], we assumed that death would almost always be considered the most worrisome outcome. We described symptomatic outcomes in lay language with expected severities based on input from clinicians and our patient and caregiver co-investigators. We described expected severities in order to decrease cognitive burden, so respondents would not need to consider probabilities. For example, we chose a mild scenario of a myocardial infarction, a mild to moderate scenario for stroke, and a severe scenario for chronic kidney disease (outcome descriptions in Additional file 1). We did not specify which outcomes were side effects from medications and which were outcomes related to hypertension.

Researchers at Johns Hopkins University pilot tested the questionnaires with our patient and caregiver co-investigators in order to assess whether the instructions, the descriptions of outcomes and the best-worst scaling tasks were clear and understandable.

### Data collection on respondent characteristics

We asked about selected respondent characteristics that could not be reliably drawn from their medical records and that we thought might influence their preferences.

We abstracted information on specific conditions from the Kaiser Permanente Virtual Data Warehouse (definitions listed in Additional file 2: Table S1), and calculated an updated Quan score [14] for the time from September 2014 to August 2016.

### Analysis

All analyses were preplanned and performed using R version 3.3.1 unless stated otherwise. Best-worst scaling surveys can be analyzed in several ways [17, 20], therefore we used three different analyses in order to suggest how to weigh different outcomes related to hypertension. The main analysis was conditional logit regression, because this is based in random utility theory and therefore real-world choice behaviour [17] and can be used to calculate utilities based on econometric models [21] (although utility is sometimes only used to refer to preference elicitation under uncertainty). In sensitivity analyses, we compared this to mean best-minus-worst scores and surface under the cumulative ranking curve (SUCRA) scores. Best-minus-worst scores are simple count scores and can be calculated for each individual – thus, they also lend themselves to explore variability and potential associations with baseline characteristics. SUCRA scores are interesting because they have a natural scale from 0 to 1 and can be therefore readily used as weights, for example in quantitative benefit-harm assessments [22, 23]. Furthermore, because both the mean best-minus-worst scores and the SUCRA scores lie in a closed range (but conditional logit parameters can be infinite), their minimum and maximum scores can indicate whether an outcome is not worrisome (i.e. most respondents choose the outcome always as least worrisome) or whether an outcome dominates (i.e. most respondents choose the outcome always as most worrisome).

In the conditional logit regression, the model outcome was defined as − 1 if it was the most worrisome outcome and + 1 if it was the least worrisome outcome, with strata defined by respondent and block. We set the least worrisome outcome as a reference so that all conditional logit coefficients were positive relative to the reference, with higher values indicating more worrisome outcomes.

Best-minus-worst scores count how many times an outcome was selected as best (least worrisome) or worst (most worrisome), averaged across respondents. The range of scores was − 5 to 5, as each outcome appeared in five of eleven blocks.

We calculated SUCRA scores using STATA version 13.1 based on estimated mean differences of the best-minus-worst scores between outcomes using a network meta-analysis model. The cumulative ranking curve of each outcome describes the probability an outcome has a certain rank or a higher one. If an outcome was always ranked as the least worrisome, it would receive a SUCRA score of 0, if it was always ranked as the most worrisome, it would receive a score of 1. The analysis is analogous to a network meta-analysis: Each block represents a trial, and each outcome in a block represents a treatment arm. The methodology was originally developed to rank treatments in a network meta-analysis of clinical trials [24]. The SUCRA analysis considered only the best-minus-worst scores of outcomes that were chosen as least or most worrisome [22]. As not choosing an outcome is also informative about the rank, the analysis could be considered as less powered than the other scores. While SUCRA scores directly reflect differences in the probability of choosing an outcome, conditional logit parameters need to be transformed for this purpose [17, 21].

To assess the variability in the preferences, we calculated individual best-minus-worst scores. Furthermore, to explore potential associations of preferences with baseline characteristics, we performed pre-planned (hypothesis-driven) subgroup analyses and (preference data-driven) hierarchical cluster analysis. Subgroup analyses were according to age, current antihypertensive treatment (yes/no), Quan adaptation of Elixhauser comorbidity index [14], self-reported number of pills per day, and self-reported life-expectancy stratified by age. We performed hierarchical cluster analysis using a variant of Ward’s minimum variance criterion that uses Euclidean distances [25]. This kind of cluster analysis defines clusters such that the variance of choices within the clusters of respondents is minimized – i.e. respondents within a cluster gave similar answers. We selected the number of clusters such that at least 25 respondents were included in each cluster.

We excluded respondents with more than two missing or invalid choices. The answer to each block counted as two choices (least and most worrisome). We counted choices as invalid if people chose more than one outcome as least or most worrisome or if they chose the same outcome as most and least worrisome. We counted choices as missing if people did not choose a least or most worrisome outcome.

Sometimes the other choices a respondent made indicated a consistent ranking that allowed us to assign the missing choice. If no such ranking could be deduced, or if the choices were inconsistent (for example outcome A is more worrisome than B, B more worrisome than C, and C more worrisome than A), the choice was assigned according to what the respondent with the most similar choices had selected.

In order to investigate whether the survey was well understood, we analyzed overall consistency of the respondents’ answers by calculating a measure of variance in best-minus-worst scores, namely the sum of the squares of each score, per respondent across outcomes [20]. The higher the variance, the more consistent the answer: If respondents answered with full consistency, one outcome had a score of − 5, and another had a score of + 5. Other outcomes had scores between − 3 and 3. If, however, respondents were not sure about how the outcomes should be ranked, the outcomes had more similar scores. Accordingly, the variance of the scores was higher if the outcomes were ranked consistently, than if they were not. The analysis of consistency considered only respondents without missing or invalid choices, as the way we replaced missing choices was expected to increase consistency. Inconsistent answers could imply either that the survey was not fully understood, or else, that the respondent perceived outcomes to be similarly worrisome. Typically, skewed distributions are observed from best-worst scaling tasks which are consistent with a gamma or a log-normal distribution – thus, most respondents answer with high consistency [20].

## Results

### Respondent characteristics

**Fig. 1:**
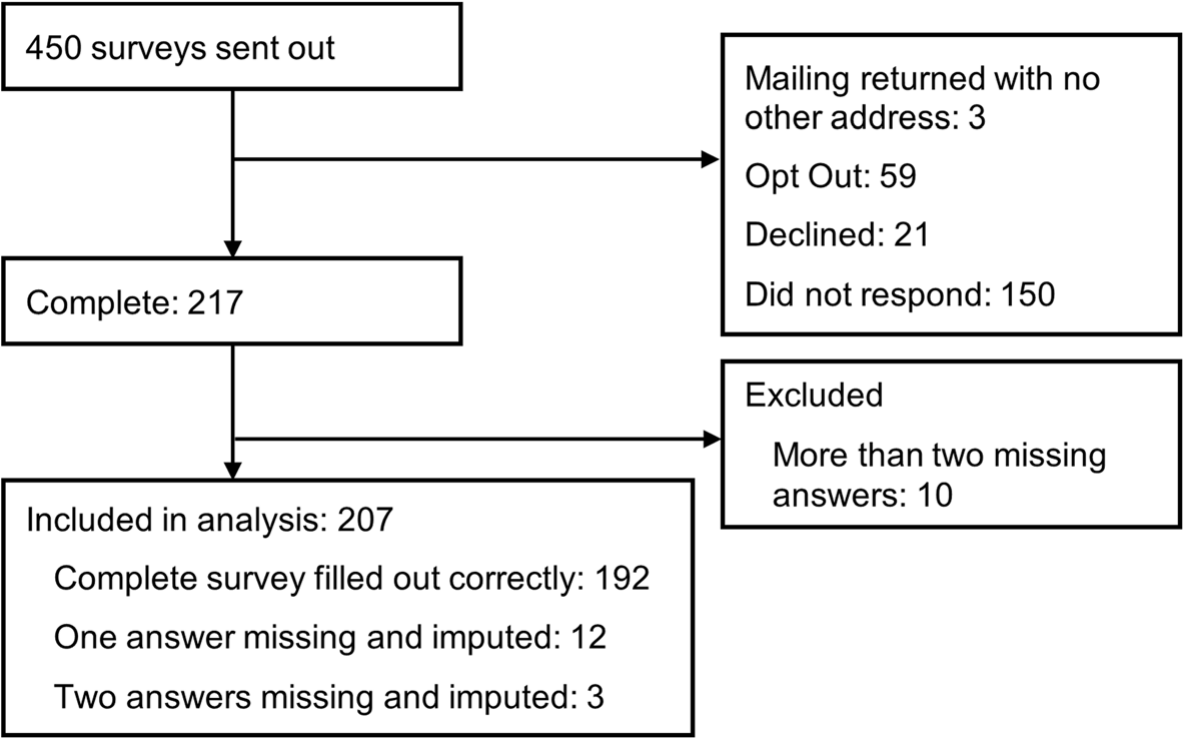
Study flow of respondents and non-respondents

Figure 1 shows the flow of respondents and non-respondents. We received 217 (out of 450) completed surveys (response rate of 48%). 192 responses were complete, and 15 were complete except for one or two missing choices. We excluded 10 respondents who had more than two missing choices (8 of 10 had 16 to 22 out of 22 choices missing, 1 had 3 and 1 had 4 missing choices), yielding a total analytic sample of 207.

Most respondents answered with high consistency (Additional file 3: Figure S1). Respondents were similar to non-respondents in terms of age, Quan score, gender distribution, race and ethnicity (Additional file 3: Table S2). Characteristics of respondents are shown in Table 1 and Table 2. Respondents were between 60 and 97 years old, mostly non-hispanic and white, and women and men were approximately equally represented. The most frequent conditions additionally to hypertension were hyperlipidemia, chronic kidney disease (stage 3 or higher) and diabetes (type II). While all respondents were hypertensive, only 76.5% were prescribed antihypertensives.

**Table 1:** Baseline characteristics of survey respondents extracted from medical records of all 217 respondents

Mean age (SD) [range]	74.5 (8.4) [60,97]
Females (%)	49.8
Race (%)	
Asian	1.8
Black/African American	3.7
White	84.8
Hawaiian/Pacific Islander	0
Native American	0
Multiple	0.5
Other	5.1
Unknown	4.1
Ethnicity (%)	
Hispanic	7.8
Non-hispanic	91.2
Unknown	0.9
Medical history (%)	
Hypertension	100
Hyperlipidemia	81.1
Diabetes mellitus type 2	41.0
Chronic kidney disease	49.8
Mild cognitive impairment	12.4
Stroke	3.2
Myocardial infarction	12.0
Congestive heart failure	15.7
Depression	32.3
Mean Total Quan Score (SD) [range]	6.1 (2.7) [3, 16]
Antihypertensives (%)	76.5
Antihyperglycemics (%)	24.4
Lipid lowering medications (%)	55.8
Smoking status (%)	
Current	6.0
Former	47.0
Never	47.0
Mean BMI (SD)	29.4 (6.2)

Chronic kidney disease was defined as stage 3 or higher, excluding end-stage renal disease. Diagnoses are listed in Additional file 2

**Table 2:** Self-reported baseline characteristics of survey respondents of all 217 respondents

Living alone (%)	25.8
Had an injurious fall in the past 12 months (%)	26.3
Has experienced low blood pressure with dizziness (%)	22.1
Has experienced passing out or fainting (%)	12.0
Currently receives dialysis (%)	1.4
Age at first treatment for high blood pressure? (%)	
30 or younger	7.4
31–45	13.8
46–60	30.9
61–75	17.5
Older than 75	5.1
Never / not anymore	6.5
Missing	18.9
Unsure	18.0
Number of pills per day currently taken (%)	
Less than 4	31.8
4–7	33.2
8–11	16.6
12–15	10.1
16–19	3.2
More than 19	3.2
Self-reported life-expectancy to age range (%)	
65–70	1.8
70–75	5.5
75–80	11.1
80–85	23.0
85–90	33.6
90 or older	24.9

For age at first high blood pressure treatment, many respondents wrote a question mark next to the age, summarized as “unsure”. Self-reported life-expectancy should be interpreted bearing in mind that many respondents were already older than the lowest proposed category

Conditional logit parameters, mean best-minus-worst scores and SUCRA scores were all similar when excluding respondents who had one or two missing choices (*n* = 15) as when including them (Additional file 3: Table S3).

### Ranking of outcomes in the study population

In the main analysis (conditional logit regression), stroke was ranked as the most worrisome outcome, followed by heart attack and heart failure (Table 3). The least worrisome outcome was treatment burden. In sensitivity analyses using mean best-minus-worst scores and SUCRA scores, ranking of outcomes was similar, but not completely identical. Across all analyses, stroke was always ranked as the most worrisome outcome; heart attack and heart failure were always the second or third most worrisome outcome; and low blood pressure with dizziness, fainting, injurious falls and treatment burden were ranked as the four least worrisome outcomes. The mean values and standard errors (Table 3) imply that while some outcomes were more worrisome than others with statistical significance, some outcomes were not ranked differently: for example, heart attack and heart failure were similarly worrisome in all analyses.

**Table 3:** Ranking of outcomes in the study population

	Main analysis		Sensitivity analysis 1		Sensitivity analysis2	
	Conditional logit parameters		Best-minus-worst scores		SUCRA scores	
Outcomes	Mean	SE	Mean	SE	Mean	SE
Stroke	3.19	0.09	−2.63	0.12	0.989	0.002
Heart attack	2.75	0.09	−1.91	0.13	0.800	0.014
Heart failure	2.73	0.09	−2.04	0.13	0.800	0.010
End stage renal disease (Dialysis)	1.95	0.09	−0.68	0.15	0.571	0.014
Cognitive impairment	1.92	0.09	−0.54	0.15	0.562	0.003
Chronic kidney disease	1.87	0.08	−0.60	0.10	0.664	0.009
Acute kidney injury	1.38	0.08	0.07	0.08	0.434	0.017
Fainting	0.44	0.08	1.63	0.11	0.117	0.016
Injurious fall	0.09	0.08	2.16	0.14	0.232	0.015
Low blood pressure with dizziness	0.06	0.08	2.18	0.11	0.08	0.03
Treatment burden	0^a^		2.35	0.14	0.253	0.007

Parameters from conditional logit regression (on log scale) with treatment burden as a reference (main analysis), mean best-minus-worst scores with a possible range of [−5,5] (sensitivity analysis 1), SUCRA scores with a possible range of [0,1] (sensitivity analysis 2).

^a^Treatment burden was chosen as the reference, as it was the least worrisome outcome, so all parameters would be positive

*SE* Standard error, *SUCRA* Surface under the cumulative ranking curve

Mean best-minus-worst scores across the study population roughly lied in the middle half of the scale, indicating that all outcomes were somewhat worrisome, and no outcome completely dominated, i.e. no outcome was always chosen as the most worrisome. SUCRA scores showed similar results. While here, stroke was the most worrisome outcome across the population, with a SUCRA score close to the maximum of the scale, the least worrisome outcome, in this case low blood pressure with dizziness, was not as close to the minimum of the scale.

### Variability of preferences between individuals

**Fig. 2:**
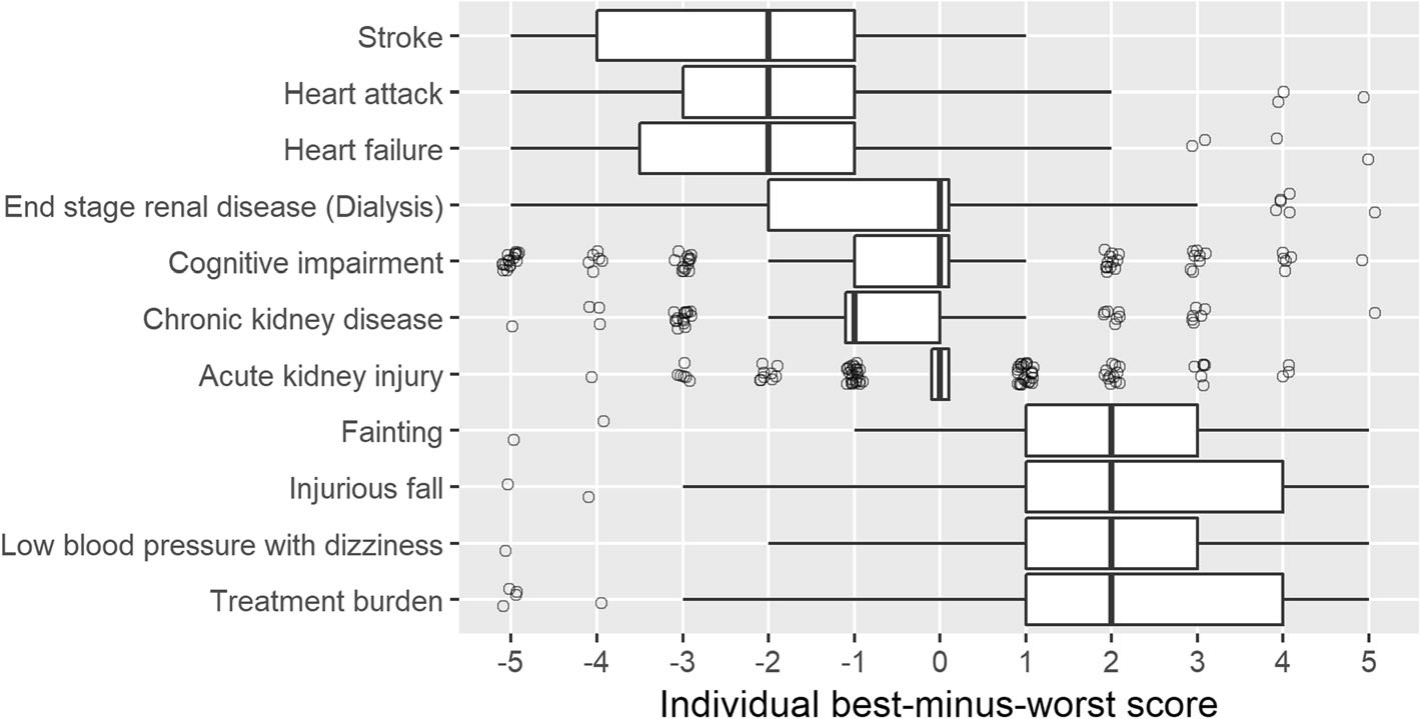
Tukey boxplot of individual best-minus-worst scores of individual respondents. Outliers are shown as circles

The range of individual best-minus-worst scores was wide (Fig. 2). In the interquartile range (IQR) for stroke, a best-minus-worst score of − 5 was not included (only 21% of the respondents always chose stroke as the most worrisome outcome). Similarly, only 18% of the respondents always chose treatment burden as the least worrisome outcome, and thus the IQR did not include 5, indicating that none of the outcomes was not worrisome in this population. While some respondents found treatment burden only a little or not worrisome, others found it more worrisome than other outcomes.

In subgroup analyses according to age, life-expectancy, number of pills per day, taking antihypertensives, and Quan score, differences in preferences were only small and not meaningful (Additional file 3: Figures S2–S6).

**Fig. 3:**
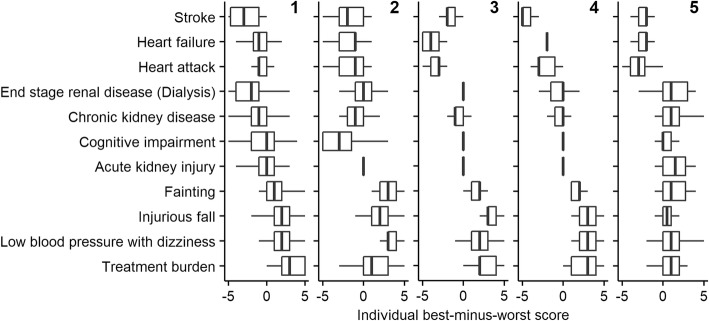
Cluster analysis of individual best-minus-worst scores. Tukey boxplots of best-minus-worst scores of individual respondents split into clusters with smaller within-cluster variance. Outliers are not shown for better readability. The number of the plot corresponds to the numbering of the clusters

Cluster analysis identified groups of respondents who made similar choices, and scored outcomes more similarly, with smaller ranges compared to Fig. 2. Different patterns were apparent (Fig. 3): The largest cluster (cluster 1, *n* = 66 / 32%) worried most about stroke, and worried more about end-stage renal disease than respondents in other clusters. Respondents of cluster 2 (*n* = 35 / 17%) worried most about cognitive impairment. Respondents of cluster 3 (*n* = 49 / 24%) worried most about heart failure, and those of cluster 4 (*n* = 31 / 15%) about stroke. Respondents of cluster 5 (*n* = 26 / 13%) worried less about kidney outcomes than other respondents, and more about treatment burden. Differences in baseline characteristics between clusters are shown in Additional file 3: Table S4.

## Discussion

Our survey showed that people with MCC and hypertension perceived stroke as the most worrisome outcome and treatment burden, low blood pressure with dizziness, injurious falls and fainting as least worrisome outcomes. Although we found differences between preferences for the eleven outcomes, our analyses indicated that the less worrisome outcomes remained relevant outcomes nevertheless. Thus, all outcomes included in this survey should be considered in population-level decisions, such as guideline development and health economic assessments, concerning people with multiple chronic conditions and hypertension, and our results could be used to define weights to balance benefits against harms of interventions.

While outcomes related to hypertension were on average considered more worrisome than adverse events related to antihypertensive therapy, our results imply that the difference in relative importance of the outcomes is not very large, and that the least worrisome outcomes were at least somewhat worrisome and should not be neglected for decision-making.

We found that while preferences varied between individuals, certain patterns could be identified using cluster analysis. For example, some patients were more worried about cognitive impairment than others. Differences in baseline characteristics between clusters were not conclusive. When cluster analyses do not identify specific patient groups, their value may be limited. However, the cluster analyses suggested common patterns of preferences that highlight the importance of decision-making: Clinicians should be aware that there are different patterns of preferences, but as they could not be attributed to specific baseline characteristics, discussing the preferences and goals with the patient is crucial.

Our subgroup analyses did not indicate associations with age, self-reported life-expectancy, antihypertensive treatment, number of medications and number of conditions (Quan score), but they may have been insufficiently powered. While one study found that older people were less willing to take an additional antihypertensive drug [11], another study did not find associations with age, nor with educational level, cognitive function, functional autonomy, information-seeking or decision-making preferences [13].

For a valid preference elicitation, it is crucial that the instrument is well understood by the study population [6], which may be challenging in older adults, especially when mild cognitive impairment is prevalent. The best-worst scaling survey was well understood, since there were few missing answers and there was high consistency. In comparison, in other, more complex hypertension preference surveys, 20–30% were unable to decide which response to choose [13]. A standard gamble exercise (where respondents have to choose between a “sure option” of a certain health state for a defined time and a “gamble option” with a defined probability of perfect health or immediate painless death) was perceived as more difficult as the health state became worse, and some found it frustrating [10]. While our response rate of 48% was relatively high considering the target population, it is possible that there could be a response bias, although comparisons between respondents and non-respondents did not show significant differences in age, sex, race, ethnicity and Quan score. We conclude that best-worst scaling is a more appropriate, feasible method for eliciting preferences in older people with MCC.

We performed the survey specifically in people with MCC, because treatment decisions and guideline development are much more challenging in this population, and less information exists about preferences for people with MCC. Age was shown to influence preference in one study [11], and age correlates with the prevalence of MCC. It is important to elicit preferences directly in the target population [6], i.e. preferences may be different in people without MCC. Guideline developers from the Kaiser Permanente National Hypertension Guideline Development Team who answered the same survey worried less about treatment burden (Additional file 3: Figure S7), confirming the importance of eliciting patient preferences. The Kaiser Permanente Colorado member population of individuals over age 65 largely reflects the demographics of the Denver Metropolitan Area, and has a similar burden of MCC as elsewhere in the US. As preference surveys across different cultures have shown little variation when cost was not included [22, 26], the outcome preferences elicited in this survey may also apply to other populations of older people with MCC and hypertension.

Our results must be interpreted in the light of the fact that we did not include death as an outcome. Based on another study, we assumed that death would almost always be considered the worst outcome [19]. Thus, including death would have led to eliciting much less information about which are the most worrisome outcomes after death.

## Conclusions

This is the first study to elicit preferences for patient-important outcomes related to hypertension among older people with MCC. The results of this study may inform population-level decisions, such as clinical practice guidelines and health economic assessments, performed for older adults with hypertension and MCC. The range of patient preferences we observed indicates that even though stroke was the most worrisome outcome, all these outcomes are important to people with multiple chronic conditions and should be considered in population-level decisions.

## Supplementary information

**Additional file 1.** Survey including outcome descriptions.

**Additional file 2.** Diagnoses used for medical history or comorbidities.

**Additional file 3.** Additional results.

## Data Availability

The data that support the findings of this study were created in a manner that is consistent with human subject protection and HIPAA privacy regulations. Source data are not publically available as they are the property of Kaiser Permanente. Survey response data may be requested from the authors and these requests will undergo internal regulatory review.

## Abbreviations

IQR: Interquartile range
MCC: Multiple chronic conditions
SE: Standard error
SUCRA: Surface under the cumulative ranking curve

## References

[1] Boyd CM, Darer J, Boult C, Fried LP, Boult L, Wu AW (2005). Clinical practice guidelines and quality of Care for Older Patients. Jama.

[2] Bennett WL, Robbins CW, Bayliss EA, Wilson R, Tabano H, Mularski RA, et al. Engaging stakeholders to inform clinical practice guidelines that address multiple chronic conditions. J Gen Intern Med. 2017:1–8. 10.1007/s11606-017-4039-5 PMC5515786 28349409

[3] Aschmann Hélène E, Boyd Cynthia M, Robbins Craig W, Mularski Richard A, Chan Wiley V, Sheehan Orla C, Wilson Renée F, Bennett Wendy L, Bayliss Elizabeth A, Yu Tsung, Leff Bruce, Armacost Karen, Glover Carol, Maslow Katie, Mintz Suzanne, Puhan Milo A (2019). Balance of benefits and harms of different blood pressure targets in people with multiple chronic conditions: a quantitative benefit-harm assessment. BMJ Open.

[4] Aschmann HE, Boyd CM, Robbins CW, Chan WV, Mularski RA, Bennett WL, et al. Informing patient-centered care through stakeholder engagement and highly stratified quantitative benefit-harm assessments. Value Health. Accepted. 10.1016/j.jval.2019.11.007 32389227

[5] Schünemann, Holger Brożek J, Guyatt G, Oxman A. GRADE Handbook. 2013. Available from: http://gdt.guidelinedevelopment.org/app/handbook/handbook.html.

[6] Zhang Yuan, Alonso-Coello Pablo, Guyatt Gordon H., Yepes-Nuñez Juan José, Akl Elie A., Hazlewood Glen, Pardo-Hernandez Hector, Etxeandia-Ikobaltzeta Itziar, Qaseem Amir, Williams John W., Tugwell Peter, Flottorp Signe, Chang Yaping, Zhang Yuqing, Mustafa Reem A., Rojas María Ximena, Schünemann Holger J. (2019). GRADE Guidelines: 19. Assessing the certainty of evidence in the importance of outcomes or values and preferences—Risk of bias and indirectness. Journal of Clinical Epidemiology.

[7] Zhang Yuan, Coello Pablo Alonso, Guyatt Gordon H., Yepes-Nuñez Juan Jose, Akl Elie A., Hazlewood Glen, Pardo-Hernandez Hector, Etxeandia-Ikobaltzeta Itziar, Qaseem Amir, Williams John W., Tugwell Peter, Flottorp Signe, Chang Yaping, Zhang Yuqing, Mustafa Reem A., Rojas María Ximena, Xie Feng, Schünemann Holger J. (2019). GRADE guidelines: 20. Assessing the certainty of evidence in the importance of outcomes or values and preferences—inconsistency, imprecision, and other domains. Journal of Clinical Epidemiology.

[8] Puhan MA, Yu T, Stegeman I, Varadhan R, Singh S, Boyd CM (2015). Benefit-harm analysis and charts for individualized and preference-sensitive prevention: example of low dose aspirin for primary prevention of cardiovascular disease and cancer. BMC Med.

[9] Zhang Y, Coello PA, Brozek J, Wiercioch W, Etxeandia-Ikobaltzeta I, Akl EA (2017). Using patient values and preferences to inform the importance of health outcomes in practice guideline development following the GRADE approach. Health Qual Life Outcomes.

[10] Weiss MC, Montgomery AA, Fahey T, Peters TJ (2004). Decision analysis for newly diagnosed hypertensive patients: a qualitative investigation. Patient Educ Couns.

[11] De Vries ST, De Vries FM, Dekker T, Haaijer-Ruskamp FM, De Zeeuw D, Ranchor AV (2015). The role of patients’ age on their preferences for choosing additional blood pressure-lowering drugs: a discrete choice experiment in patients with diabetes. PLoS One.

[12] McAlister FA, O’Connor AM, Wells G, Grover SA, Laupacis A (2000). When should hypertension be treated? The different perspectives of Canadian family physicians and patients. CMAJ.

[13] Perret-Guillaume C, Genet C, Herrmann FR, Benetos A, Hurst SA, Vischer UM (2011). Attitudes and approaches to decision making about antihypertensive treatment in elderly patients. J Am Med Dir Assoc.

[14] Quan H, Sundararajan V, Halfon P, Fong A, Burnand B, Luthi JC (2005). Coding algorithms for defining comorbidities in ICD-9-CM and ICD-10 administrative data. Med Care.

[15] Ross Tyler R., Ng Daniel, Brown Jeffrey S., Pardee Roy, Hornbrook Mark C., Hart Gene, Steiner John F. (2014). The HMO Research Network Virtual Data Warehouse: A Public Data Model to Support Collaboration. eGEMs (Generating Evidence & Methods to improve patient outcomes).

[16] Flynn TN, Louviere JJ, Peters TJ, Coast J (2007). Best-worst scaling: what it can do for health care research and how to do it. J Health Econ.

[17] Mühlbacher AC, Kaczynski A, Zweifel P, Johnson FR (2016). Experimental measurement of preferences in health and healthcare using best-worst scaling: an overview. Health Econ Rev.

[18] Finn A, Louviere JJ (1992). Determining the appropriate response to evidence of public concern: the case of food safety. J Public Policy Mark.

[19] Stafinski Tania, Menon Devidas, Nardelli Alex, Bakal Jeff, Ezekowitz Justin, Tymchak Wayne, Welsh Robert, Gyenes Gabor, Armstrong Paul W. (2015). Incorporating patient preferences into clinical trial design: Results of the Opinions of Patients on Treatment Implications of New Studies (OPTIONS) project. American Heart Journal.

[20] Louviere JJ, Flynn TN, Marley AA. Best-worst scaling: theory, methods and applications. Cambridge: Cambridge University Press; 2015.

[21] Severin F, Schmidtke J, Mühlbacher A, Rogowski WH (2013). Eliciting preferences for priority setting in genetic testing: a pilot study comparing best-worst scaling and discrete-choice experiments. Eur J Hum Genet.

[22] Yebyo HG, Aschmann HE, Yu T, Puhan MA (2018). Should statin guidelines consider patient preferences? Eliciting preferences of benefit and harm outcomes of statins for primary prevention of cardiovascular disease in the sub-Saharan African and European contexts. BMC Cardiovasc Disord.

[23] Yebyo Henock G., Aschmann Hélène E., Puhan Milo A. (2018). Finding the Balance Between Benefits and Harms When Using Statins for Primary Prevention of Cardiovascular Disease. Annals of Internal Medicine.

[24] Salanti G, Ades AE, Ioannidis JPA (2011). Graphical methods and numerical summaries for presenting results from multiple-treatment meta-analysis: an overview and tutorial. J Clin Epidemiol.

[25] Murtagh F, Legendre P (2014). Ward’s hierarchical agglomerative clustering method: which algorithms implement Ward’s criterion?. J Classif.

[26] Salomon JA, Vos T, Hogan DR, Gagnon M, Naghavi M, Mokdad A (2012). Common values in assessing health outcomes from disease and injury: disability weights measurement study for the global burden of disease study 2010. Lancet.

