# Supplementary material for: Outcome preferences of older people with multiple chronic conditions and hypertension: a cross-sectional survey using best-worst scaling
Source: Health Qual Life Outcomes. 2019 Dec 19;17:186. doi: 10.1186/s12955-019-1250-6 (PMC6924040; doi:10.1186/s12955-019-1250-6)
Supplement: Supplementary file 1 — Additional file 1. Survey including outcome descriptions. [file 12955_2019_1250_MOESM1_ESM.docx]

Additional File 1: Survey including outcome descriptions

Outcome preferences of older people with multiple chronic conditions and hypertension: A cross-sectional survey using best-worst scaling

Hélène E Aschmann, Milo A Puhan, Craig W Robbins, Elizabeth A Bayliss, Wiley V Chan, Richard A Mularski, Renee F Wilson, Wendy L Bennett, Orla C Sheehan, Tsung Yu, Henock G Yebyo, Bruce Leff, Heather Tabano, Karen Armacost, Carol Glover, Katie Maslow, Suzanne Mintz, Cynthia M Boyd

**Survey Questions**

Below is a situation that may occur for some people with high blood pressure. Pretend that this situation is happening to you and then please answer the survey questions as though you were in this situation. Each question will ask you to choose which of five health problems would be most and least concerning to you. Most of these health problems can have a range of severity from more to less severe. Please refer to the Definition of Health Problems on page 8 to help you choose. There are no right or wrong answers. The same situation applies to all of the questions.

| **Possible Situation** |
| --- |
| *Imagine that your doctor tells you that your blood pressure level is too high. Your doctor recommends that you add another medication to lower your blood pressure. The additional medicine may affect your risk for some health problems. To help guide your blood pressure treatment, your doctor wants to know which health problem would be most worrisome to you and which would be least worrisome.* |

*****Example Survey Question*****

*If one of the following health problems happened to you, which would worry you most and which would worry you least?*

*Please indicate:*

- *The health problem that would worry you most (check only one)*
- *The health problem that would worry you least (check only one)*

| ***Problem*** | ***Most worrisome***  *(choose one)* | ***Least worrisome***  *(choose one)* |
| --- | --- | --- |
| *Acute kidney injury* |  |  |
| *Low blood pressure with dizziness* |  |  |
| *Fall* |  |  |
| *Cognitive Impairment* |  |  |
| *Heart failure* |  |  |

***The person who answered this question would be most worried about “heart failure” and least worried about “low blood pressure with dizziness”.***

1. **For each question, please indicate which health problem would worry you the most and which would worry you the least.**

**Q1.** If one of the following health problems were to happen to you, which would worry you most and which would worry you least?

| **Problem** | **Most worrisome**  (choose one) | **Least worrisome**  (choose one) |
| --- | --- | --- |
| Heart failure |  |  |
| Stroke |  |  |
| Dialysis |  |  |
| Fall |  |  |
| Acute kidney injury |  |  |

**Q2.** If one of the following health problems were to happen to you, which would worry you most and which would worry you least?

| **Problem** | **Most worrisome**  (choose one) | **Least worrisome**  (choose one) |
| --- | --- | --- |
| Acute kidney injury |  |  |
| Heart attack |  |  |
| Low blood pressure with dizziness |  |  |
| Stroke |  |  |
| Passing out or fainting |  |  |

**Q3.** If one of the following health problems were to happen to you, which would worry you most and which would worry you least?

| **Problem** | **Most worrisome**  (choose one) | **Least worrisome**  (choose one) |
| --- | --- | --- |
| Chronic kidney disease |  |  |
| Heart failure |  |  |
| Stroke |  |  |
| More treatment burdens |  |  |
| Heart attack |  |  |

**Q4.** If one of the following health problems were to happen to you, which would worry you most and which would worry you least?

| **Problem** | **Most worrisome**  (choose one) | **Least worrisome**  (choose one) |
| --- | --- | --- |
| Stroke |  |  |
| More treatment burdens |  |  |
| Cognitive impairment |  |  |
| Low blood pressure with dizziness |  |  |
| Dialysis |  |  |

**Q5.** If one of the following health problems were to happen to you, which would worry you most and which would worry you least?

| **Problem** | **Most worrisome**  (choose one) | **Least worrisome**  (choose one) |
| --- | --- | --- |
| Low blood pressure with dizziness |  |  |
| Dialysis |  |  |
| Chronic kidney disease |  |  |
| Heart attack |  |  |
| Fall |  |  |

**Q6.** If one of the following health problems were to happen to you, which would worry you most and which would worry you least?

| **Problem** | **Most worrisome**  (choose one) | **Least worrisome**  (choose one) |
| --- | --- | --- |
| Cognitive impairment |  |  |
| Acute kidney injury |  |  |
| Heart failure |  |  |
| Chronic kidney disease |  |  |
| Low blood pressure with dizziness |  |  |

**Q7.** If one of the following health problems were to happen to you, which would worry you most and which would worry you least?

| **Problem** | **Most worrisome**  (choose one) | **Least worrisome**  (choose one) |
| --- | --- | --- |
| Fall |  |  |
| Chronic kidney disease |  |  |
| Passing out or fainting |  |  |
| Cognitive impairment |  |  |
| Stroke |  |  |

**Q8.** If one of the following health problems were to happen to you, which would worry you most and which would worry you least?

| **Problem** | **Most worrisome**  (choose one) | **Least worrisome**  (choose one) |
| --- | --- | --- |
| Dialysis |  |  |
| Cognitive impairment |  |  |
| Heart attack |  |  |
| Passing out or fainting |  |  |
| Heart failure |  |  |

**Q9.** If one of the following health problems were to happen to you, which would worry you most and which would worry you least?

| **Problem** | **Most worrisome**  (choose one) | **Least worrisome**  (choose one) |
| --- | --- | --- |
| Passing out or fainting |  |  |
| Low blood pressure with dizziness |  |  |
| Fall |  |  |
| Heart failure |  |  |
| More treatment burdens |  |  |

**Q10.** If one of the following health problems were to happen to you, which would worry you most and which would worry you least?

| **Problem** | **Most worrisome**  (choose one) | **Least worrisome**  (choose one) |
| --- | --- | --- |
| Heart Attack |  |  |
| Fall |  |  |
| More treatment burdens |  |  |
| Acute kidney injury |  |  |
| Cognitive impairment |  |  |

**Q11.** If one of the following health problems were to happen to you, which would worry you most and which would worry you least?

| **Problem** | **Most worrisome**  (choose one) | **Least worrisome**  (choose one) |
| --- | --- | --- |
| More treatment burdens |  |  |
| Passing out or fainting |  |  |
| Acute kidney injury |  |  |
| Dialysis |  |  |
| Chronic kidney disease |  |  |

1. **Additional Questions**

**Q12. Do you live alone?**

Yes

No

**Q13. Have you fallen and injured yourself in the past 12 months?**

Yes

No

**Q14. Have you experienced low blood pressure with dizziness?**

Yes

No

**Q15. Have you experienced passing out or fainting?**

Yes

No

**Q16. Do you currently receive dialysis?**

Yes

No

**Q17. At what age were you first treated for high blood pressure? __________**

**Q18. How many pills per day are you taking now?**

less than 4

4-7

8-11

12-15

16-19

more than 19

**Q19. If you were to guess, to what age would you expect to live?**

65-70

70-75

75-80

80-85

85-90

90 or older

1. **Definitions of Health Problems**

This section is for your reference and provides definitions of health problems described in the survey questions. All of these health problems can potentially be related to diabetes or to treatments for diabetes. Most of these health problems can have a range of severity from more to less severe, but please consider the definitions when you answer the questions in the survey.

| **Heart attack** | **Stroke** |
| --- | --- |
| *Suddenly you have chest pain, feel short of breath and nauseated. You are very worried and call an ambulance. After a few days in hospital and some tests and procedures you return home. You now take a number of new medicines and are making changes to your diet and exercising regularly.* | *You have sudden weakness in your left arm, difficulty walking and are slurring your words. A family member calls an ambulance. You spend a number of days in hospital and then move to a rehabilitation facility for another 2 weeks. Your left arm is still weaker than it was before the stroke but your speech is much better. You can’t drive anymore.* |
| **Acute kidney injury** | **Chronic kidney disease** |
| *Your kidneys are damaged suddenly and can’t do their job of removing waste products from your blood. You feel quite unwell and nauseated, very tired and short of breath. You are admitted to hospital for five days and your medications have changed. Now you are back at home, your kidneys are working again, pretty close to the way they were before.* | *Your kidneys can't filter your blood to remove waste products like they should. You feel tired, your appetite is poor. Your legs are swollen and you have difficulty sleeping because of leg cramps. You are on a special diet and have to watch how much protein and potassium you eat. You have to make sure that you don’t drink too much liquid. You see a kidney specialist and take a number of new medicines.* |
| **Dialysis** | **Heart failure** |
| *Your kidneys have stopped removing waste products from your blood. You need to go to the dialysis center 3 times a week and sit attached to a machine which cleans your blood for 4 hours at a time and returns clean blood back to your body. You feel tired and have to be very careful what you eat and how much liquid you drink. You have to weigh yourself every day and take lots of medicines.* | *Your heart doesn’t pump blood as well as it should. You feel tired, are short of breath and your legs are swollen. You need to take three new medicines. You have to go the bathroom many times during the day and at night.* |
| **Cognitive Impairment** | **Low blood pressure with dizziness** |
| *You have trouble remembering recent events, learning new things, concentrating, making decisions or planning something. Although you notice the symptoms and they bother you, you can still do things for yourself.* | *Your blood pressure goes too low and you feel weak and dizzy. You feel nauseous and your vision goes a bit blurry. You do not pass out, but if you don’t sit down you will fall. This happens several times a day.* |
| **Passing out or fainting** | **More treatment burdens** |
| *Before you faint you feel dizzy, lightheaded, clammy and a bit nauseous. Your vision starts to “black out” and the next thing you remember is waking up on the floor. You recover quickly but are a bit frightened by what has happened. You seek medical care and your doctor decides to change your blood pressure medicines to different ones.* | *You need to take 2 extra tablets every day to keep your blood pressure controlled. You have to get your blood pressure checked regularly and are working hard to stick to a diet and exercise plan. You have more doctor appointments than in the past.* |
| **Fall** |  |
| *You have a fall and injure your ankle. You go to the emergency department. Your ankle is broken and you need to wear a cast for a month.* |  |
