# Supplementary material for: Outcome preferences of older people with multiple chronic conditions and hypertension: a cross-sectional survey using best-worst scaling
Source: Health Qual Life Outcomes. 2019 Dec 19;17:186. doi: 10.1186/s12955-019-1250-6 (PMC6924040; doi:10.1186/s12955-019-1250-6)
Supplement: Supplementary file 2 — Additional file 2. Diagnoses used for medical history or comorbidities. [file 12955_2019_1250_MOESM2_ESM.docx]

Additional File 2: Diagnoses used for medical history or comorbidities

Outcome preferences of older people with multiple chronic conditions and hypertension: A cross-sectional survey using best-worst scaling

Hélène E Aschmann, Milo A Puhan, Craig W Robbins, Elizabeth A Bayliss, Wiley V Chan, Richard A Mularski, Renee F Wilson, Wendy L Bennett, Orla C Sheehan, Tsung Yu, Henock G Yebyo, Bruce Leff, Heather Tabano, Karen Armacost, Carol Glover, Katie Maslow, Suzanne Mintz, Cynthia M Boyd

**Table S1: Diagnoses by ICD codes**

All subsequent digits pulled where available unless otherwise noted (eg. 401 = 401.*, E78 = E78*)

| Hypertension  (Quan-Charlson definition) | 401  402  403  404  405  I10  I11.0  I11.9  I12.0  I12.9  I13.0  I13.10  I13.11  I13.2  I15.0  I15.1  I15.2  I15.8  I15.9 |
| --- | --- |
| Hyperlipidemia | 272  E78 |
| Diabetes Type 2  (Original cohort definition) | 250.x0  250.x2  E11 |
| Chronic Kidney Disease | 585.3  585.4  585.5  585.6  N18.3  N18.4  N18.5  N18.6 |
| Cognitive Impairment | 331.83  G31.84  R41.81  R41.841 |
| Myocardial Infarction  (Quan-Charlson definition) | 410  412  I21  I22  I25.2 |
| Congestive Heart Failure  (Quan-Elixhauser and Quan-Charlson definitions) | 398.91  402.01  402.11  402.91  404.01  404.03  404.11  404.13  404.91  404.93  425.4  425.5  425.6  425.7  425.8  425.9  428  I09.9  I11.0  I13.0  I13.2  I25.5  I42.0  I42.5  I42.6  I42.7  I42.8  I42.9  I43  I50  P29.0 |
| Depression  (Quan-Charlson definition) | 296.2  296.3  296.5  300.4  309  311  F20.4  F31.3  F31.4  F31.5  F32  F33  F34.1  F41.2  F43.2 |
| Stroke | 433.01  433.11  433.21  433.31  433.81  433.91  434.01  434.11  434.91  436  430  I60  I60.0  I60.00  I60.01  I60.02  I60.1  I60.10  I60.11  I60.12  I60.2  I60.3  I60.30  I60.31  I60.32  I60.4  I60.5  I60.50  I60.51  I60.52  I60.6  I60.7  I60.8  I60.9  431  I61  I61.0  I61.1  I61.2  I61.3  I61.4  I61.5  I61.6  I61.8  I61.9  163  I63.0  I63.00  I63.01  I63.011  I63.012  I63.013  I63.019  I63.02  I63.03  I63.031  I63.032  I63.033  I63.039  I63.09  I63.1  I63.10  I63.11  I63.111  I63.112  I63.113  I63.119  I63.12  I63.13  I63.131  I63.132  I63.133  I63.139  I63.19  I63.2  I63.20  I63.21  I63.211  I63.212  I63.213  I63.219  I63.22  I63.23  I63.231  I63.232  I63.233  I63.239  I63.29  I63.3  I63.30  I63.31  I63.311  I63.312  I63.313  I63.319  I63.32  I63.321  I63.322  I63.323  I63.329  I63.33  I63.331  I63.332  I63.333  I63.339  I63.34  I63.341  I63.342  I63.343  I63.349  I63.39  I63.4  I63.40  I63.41  I63.411  I63.412  I63.413  I63.419  I63.42  I63.421  I63.422  I63.423  I63.429  I63.43  I63.431  I63.432  I63.433  I63.439  I63.44  I63.441  I63.442  I63.443  I63.449  I63.49  I63.5  I63.50  I63.51  I63.511  I63.512  I63.513  I63.519  I63.52  I63.521  I63.522  I63.523  I63.529  I63.53  I63.531  I63.532  I63.533  I63.539  I63.54  I63.541  I63.542  I63.543  I63.549  I63.59  I63.6  I63.8  I63.9 |
