# Supplementary material for: Outcome preferences of older people with multiple chronic conditions and hypertension: a cross-sectional survey using best-worst scaling
Source: Health Qual Life Outcomes. 2019 Dec 19;17:186. doi: 10.1186/s12955-019-1250-6 (PMC6924040; doi:10.1186/s12955-019-1250-6)
Supplement: Supplementary file 3 — Additional file 3. Additional results. [file 12955_2019_1250_MOESM3_ESM.docx]

Additional File 3: Additional results

Outcome preferences of older people with multiple chronic conditions and hypertension: A cross-sectional survey using best-worst scaling

Hélène E Aschmann, Milo A Puhan, Craig W Robbins, Elizabeth A Bayliss, Wiley V Chan, Richard A Mularski, Renee F Wilson, Wendy L Bennett, Orla C Sheehan, Tsung Yu, Henock G Yebyo, Bruce Leff, Heather Tabano, Karen Armacost, Carol Glover, Katie Maslow, Suzanne Mintz, Cynthia M Boyd

Table S2: Characteristics of respondents and non-respondents in the survey

|  | Completed (N=217) | Not completed (N=233) |
| --- | --- | --- |
| Female | 108 (49.8%) | 129 (55.4%) |
| Race |  |  |
| White | 184 (84.8%) | 181 (77.7%) |
| Black/African American | 8 (3.7%) | 14 (6.0%) |
| Asian | 4 (1.8%) | 3 (1.3%) |
| Native American | 0 (0%) | 1 (0.4%) |
| Hawaiian/Pacific Islander | 0 (0%) | 0 (0%) |
| Multiple | 1 (0.5%) | 2 (0.9%) |
| Other | 11 (5.1%) | 10 (4.3%) |
| Unknown | 9 (4.1%) | 22 (9.4%) |
| Ethnicity |  |  |
| Hispanic | 17 (7.8%) | 35 (15.0%) |
| Non-Hispanic | 198 (91.2%) | 189 (81.1%) |
| Unknown | 2 (0.9%) | 9 (3.9%) |
| Age |  |  |
| 60-69 | 71 (32.7%) | 88 (37.8%) |
| 70-79 | 84 (38.7%) | 95 (40.8%) |
| 80-89 | 55 (25.3%) | 41 (17.6%) |
| 90-99 | 7 (3.2%) | 9 (3.9%) |
| Mean age ± SD | 74.5 ± 8.4 | 73.3 ± 8.3 |
| Quan score |  |  |
| 3-5 | 131 (60.4%) | 154 (66.1%) |
| 6-8 | 65 (30%) | 58 (24.9%) |
| 9-11 | 17 (7.8%) | 15 (6.4%) |
| 12-14 | 3 (1.4%) | 5 (2.1%) |
| 15-16 | 1 (0.5%) | 1 (0.4%) |
| Mean score ± SD | 5.3 ± 2.3 | 5.3 ± 2.3 |

Table S3: Ranking of outcomes in the study population without imputed values

|  | Main analysis | | Sensitivity analysis 1 | | | Sensitivity analysis2 | |
| --- | --- | --- | --- | --- | --- | --- | --- |
|  | Conditional logit parameters | | Mean best-minus-worst scores | | | SUCRA scores | |
| Outcomes | Mean | SE | Mean | SE | Mean | | SE |
| Stroke | 3.12 | 0.09 | -2.59 | 0.12 | 0.9876 | | 0.0018 |
| Heart attack | 2.68 | 0.09 | -1.89 | 0.13 | 0.795 | | 0.009 |
| Heart failure | 2.69 | 0.09 | -2.04 | 0.13 | 0.797 | | 0.011 |
| End stage renal disease (Dialysis) | 1.92 | 0.09 | -0.69 | 0.15 | 0.571 | | 0.007 |
| Cognitive impairment | 1.87 | 0.09 | -0.53 | 0.15 | 0.569 | | 0.013 |
| Chronic kidney disease | 1.84 | 0.09 | -0.63 | 0.10 | 0.668 | | 0.009 |
| Acute kidney injury | 1.33 | 0.09 | 0.08 | 0.09 | 0.432 | | 0.016 |
| Fainting | 0.40 | 0.08 | 1.64 | 0.11 | 0.107 | | 0.012 |
| Injurious fall | 0.04 | 0.08 | 2.17 | 0.14 | 0.230 | | 0.013 |
| Low blood pressure with dizziness | 0.02 | 0.08 | 2.19 | 0.11 | 0.08 | | 0.03 |
| Treatment burden | 0.00 | 0.00 | 2.29 | 0.15 | 0.262 | | 0.013 |

Parameters from conditional logit regression (on log scale) with treatment burden as a reference (main analysis), mean best-minus-worst scores with a possible range of [-5,5] (sensitivity analysis 1), SUCRA scores with a possible range of [0,1] (sensitivity analysis 2).
* Treatment burden was chosen as the reference, as it was the least worrisome outcome, so all parameters would be positive.
SE: Standard error, SUCRA: Surface under the cumulative ranking curve

Table S4: Baseline characteristics of respondents in different clusters

|  | Cluster 1 (n=66) | Cluster 2 (n=35) | Cluster 3 (n=49) | Cluster 4 (n=31) | Cluster 5 (n=26) |
| --- | --- | --- | --- | --- | --- |
| Baseline characteristics extracted from medical records | | | | | |
| Mean age (SD) [range] | 75 (8.3) [60,94] | 73.9 (8.9) [60,97] | 74.5 (8.4) [60,94] | 72.3 (7.9) [60,88] | 75.1 (8.2) [62,89] |
| Females (%) | 39.4 | 71.4 | 53.1 | 48.4 | 46.2 |
| Race (%) |  |  |  |  |  |
| Asian | 1.5 | 5.7 | 2.0 | 0 | 0 |
| Black/African American | 3.0 | 0 | 8.2 | 3.2 | 3.8 |
| White | 87.9 | 80.0 | 73.5 | 90.3 | 96.2 |
| Multiple | 0 | 2.9 | 0 | 0 | 0 |
| Other | 1.5 | 8.6 | 10.2 | 6.5 | 0 |
| Unknown | 6.1 | 2.9 | 6.1 | 0 | 0 |
| Ethnicity (%) | | | | | |
| Hispanic | 6.1 | 5.7 | 14.3 | 9.7 | 0 |
| Non-hispanic | 92.4 | 91.4 | 85.7 | 90.3 | 100 |
| Unknown | 1.5 | 2.9 | 0 | 0 | 0 |
| Medical history (%) | | | | | |
| Hypertension | 100 | 100 | 100 | 100 | 100 |
| Hyperlipidemia | 81.8 | 71.4 | 75.5 | 83.9 | 92.3 |
| Diabetes mellitus type 2 | 39.4 | 37.1 | 49.0 | 38.7 | 38.5 |
| Chronic kidney disease | 51.5 | 60.0 | 49.0 | 41.9 | 38.5 |
| Cognitive impairment | 13.6 | 17.1 | 12.2 | 6.5 | 7.7 |
| Stroke | 3.0 | 0 | 2.0 | 3.2 | 3.8 |
| Myocardial infarction | 10.6 | 8.6 | 18.4 | 3.2 | 23.1 |
| Congestive heart failure | 16.7 | 11.4 | 20.4 | 9.7 | 11.5 |
| Depression | 28.8 | 40.0 | 28.6 | 41.9 | 30.8 |
| Mean Total Quan Score (SD) [range] | 6.4 (2.7) [3,16] | 6.6 (3.1) [3,13] | 5.7 (2.6) [3,15] | 5.5 (2.1) [3,10] | 5.3 (2.3) [3,12] |
| Taking medication(%) | | | | | |
| Antihypertensives | 84.8 | 80.0 | 79.6 | 61.3 | 73.1 |
| Antihyperglycemics | 30.3 | 20.0 | 28.6 | 22.6 | 7.7 |
| Antihyperlipidemics | 57.6 | 42.9 | 55.1 | 64.5 | 65.4 |
| Smoking status (%) | | | | | |
| Current | 10.6 | 2.9 | 0 | 6.5 | 11.5 |
| Former | 42.4 | 45.7 | 49.0 | 51.6 | 50.0 |
| Never | 47.0 | 51.4 | 51.0 | 41.9 | 38.5 |
| Mean BMI (SD) | 30.5 (6.7) | 29.8 (6.5) | 29.4 (5) | 29.9 (6.8) | 26.5 (4.3) |
| Self-reported baseline characteristics | | | | | |
| Do you live alone? (%) | | | | | |
| Yes | 24.2 | 40.0 | 20.4 | 19.4 | 26.9 |
| No | 75.8 | 60.0 | 79.6 | 80.6 | 73.1 |
| Have you fallen and injured yourself in the past 12 months? (%) | | | | | |
| Yes | 25.8 | 31.4 | 22.4 | 29.0 | 26.9 |
| No | 74.2 | 68.6 | 77.6 | 71.0 | 73.1 |
| Have you experienced low blood pressure with dizziness? (%) | | | | | |
| Yes | 25.8 | 25.7 | 18.4 | 16.1 | 23.1 |
| No | 71.2 | 71.4 | 81.6 | 83.9 | 76.9 |
| Have you experienced passing out or fainting? (%) | | | | | |
| Yes | 12.1 | 14.3 | 10.2 | 6.5 | 19.2 |
| No | 87.9 | 85.7 | 89.8 | 93.5 | 80.8 |
| Do you currently receive diaylsis? (%) | | | | | |
| Yes | 1.5 | 0 | 2 | 0 | 0 |
| No | 98.5 | 100 | 98.0 | 100 | 100 |
| How many pills per day are you taking now? (%) | | | | | |
| Less than 4 | 27.3 | 28.6 | 38.8 | 35.5 | 30.8 |
| 4-7 | 34.8 | 40.0 | 24.5 | 41.9 | 38.5 |
| 8-11 | 15.2 | 17.1 | 20.4 | 9.7 | 11.5 |
| 12-15 | 16.7 | 5.7 | 6.1 | 9.7 | 11.5 |
| 16-19 | 1.5 | 5.7 | 6.1 | 0 | 0 |
| More than 19 | 4.5 | 2.9 | 2.0 | 0 | 3.8 |
| At what age were you first treated for high blood pressure? (%) | | | | | |
| 30 or younger | 7.6 | 14.3 | 6.1 | 6.5 | 3.8 |
| 31-45 | 18.2 | 14.3 | 14.3 | 9.7 | 7.7 |
| 46-60 | 24.2 | 34.3 | 24.5 | 32.3 | 23.1 |
| 61-75 | 9.1 | 11.4 | 10.2 | 9.7 | 11.5 |
| Older than 75 | 3.0 | 2.9 | 6.1 | 6.5 | 3.8 |
| Never / not anymore | 22.7 | 17.1 | 14.3 | 19.4 | 7.7 |
| Unsure | 1.5 | 5.7 | 6.1 | 6.5 | 19.2 |
| Missing | 21.2 | 17.1 | 14.3 | 16.1 | 23.1 |
| If you were to guess, to what age would you expect to live? (%) | | | | | |
| 65-70 | 1.5 | 5.7 | 2.0 | 0 | 0 |
| 70-75 | 6.1 | 8.6 | 2.0 | 9.7 | 3.8 |
| 75-80 | 15.2 | 11.4 | 10.2 | 9.7 | 3.8 |
| 80-85 | 13.6 | 37.1 | 22.4 | 35.5 | 19.2 |
| 85-90 | 48.5 | 20.0 | 26.5 | 22.6 | 34.6 |
| 90 or older | 15.2 | 17.1 | 36.7 | 22.6 | 38.5 |


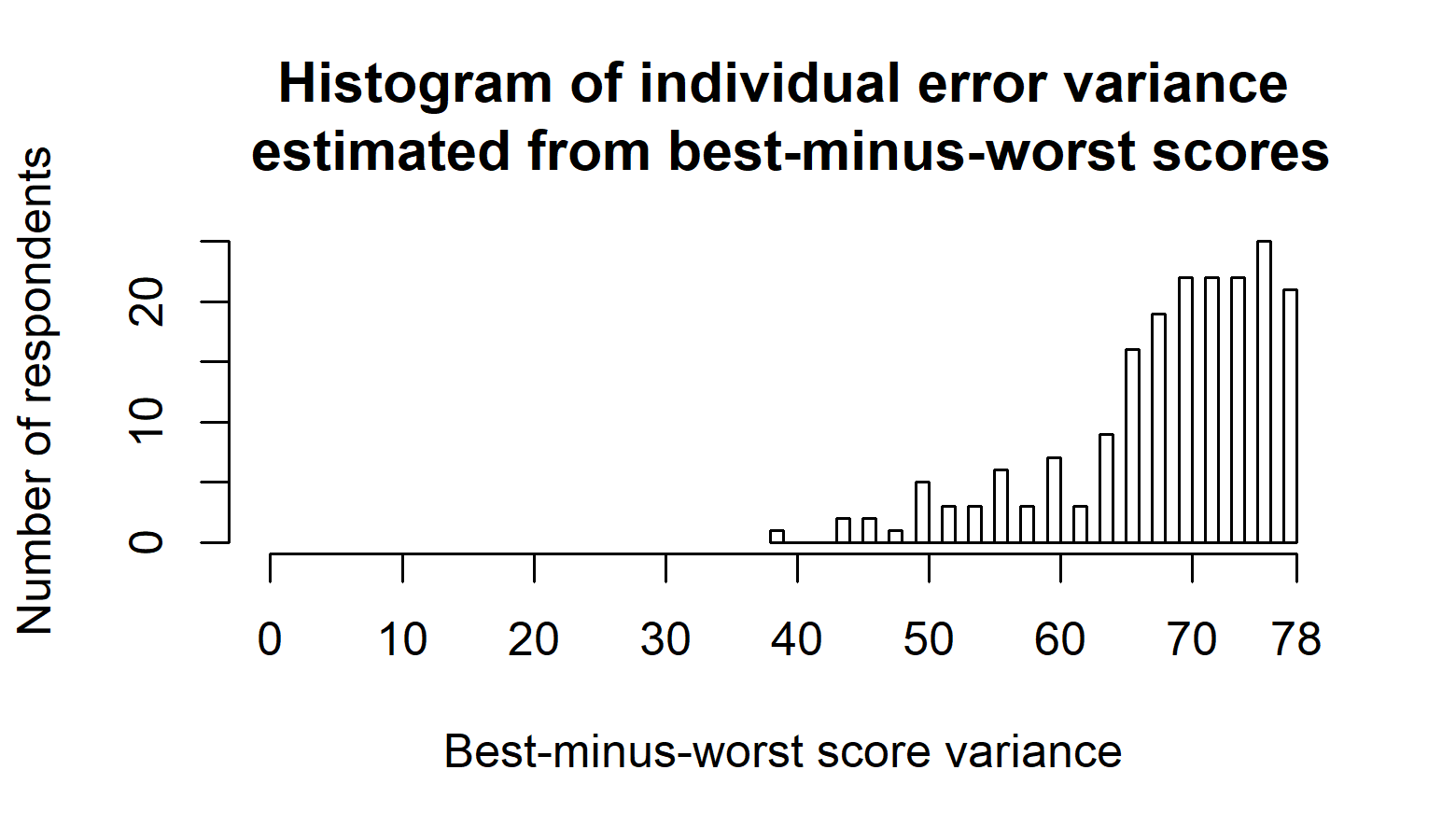


Figure S1: Histogram of the variance in best-minus-worst scores over all outcomes per person.

The higher the variance, the more consistent the answer by the respondent.


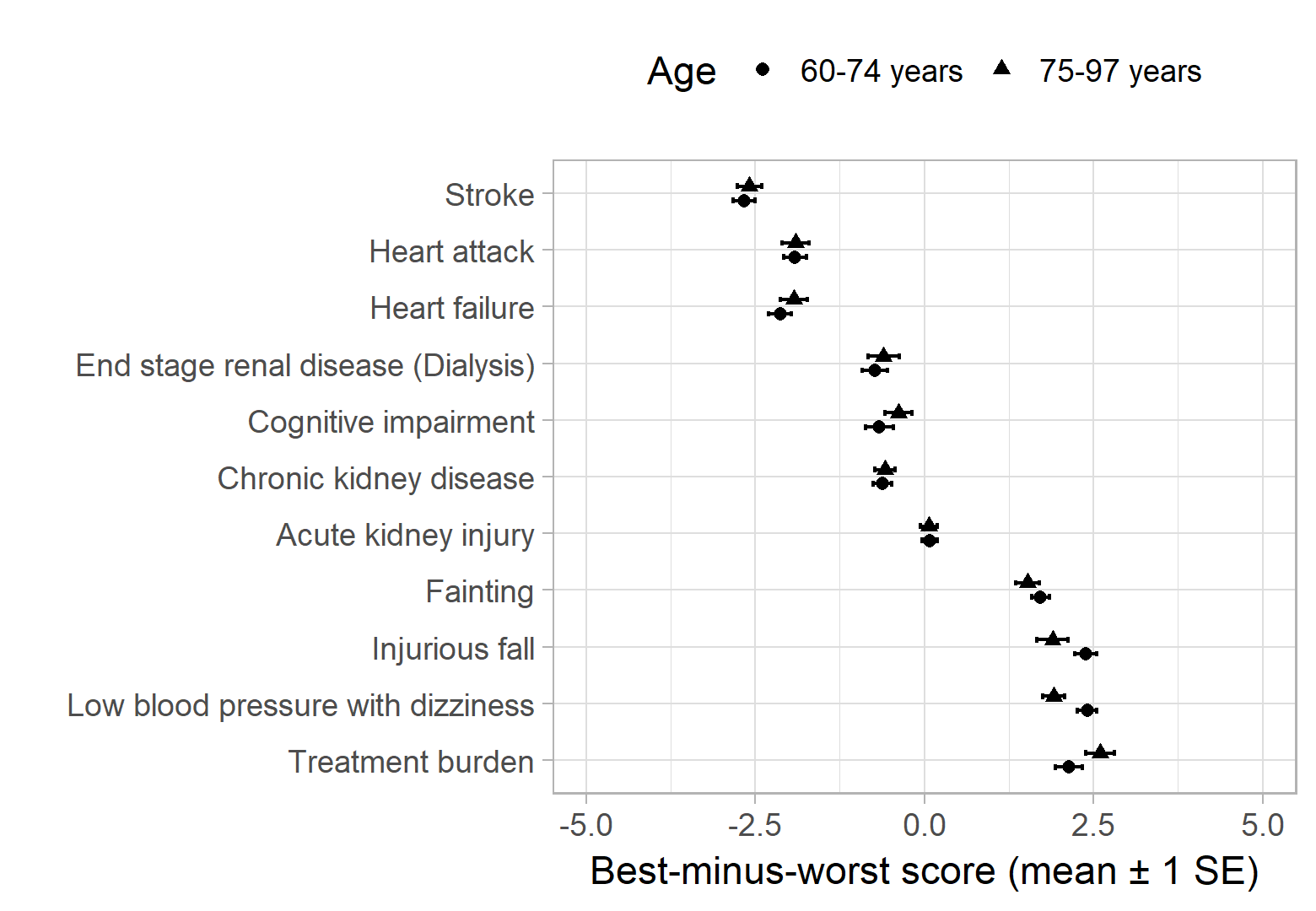


Figure S2: Subgroup analysis of best-minus-worst scores by age.


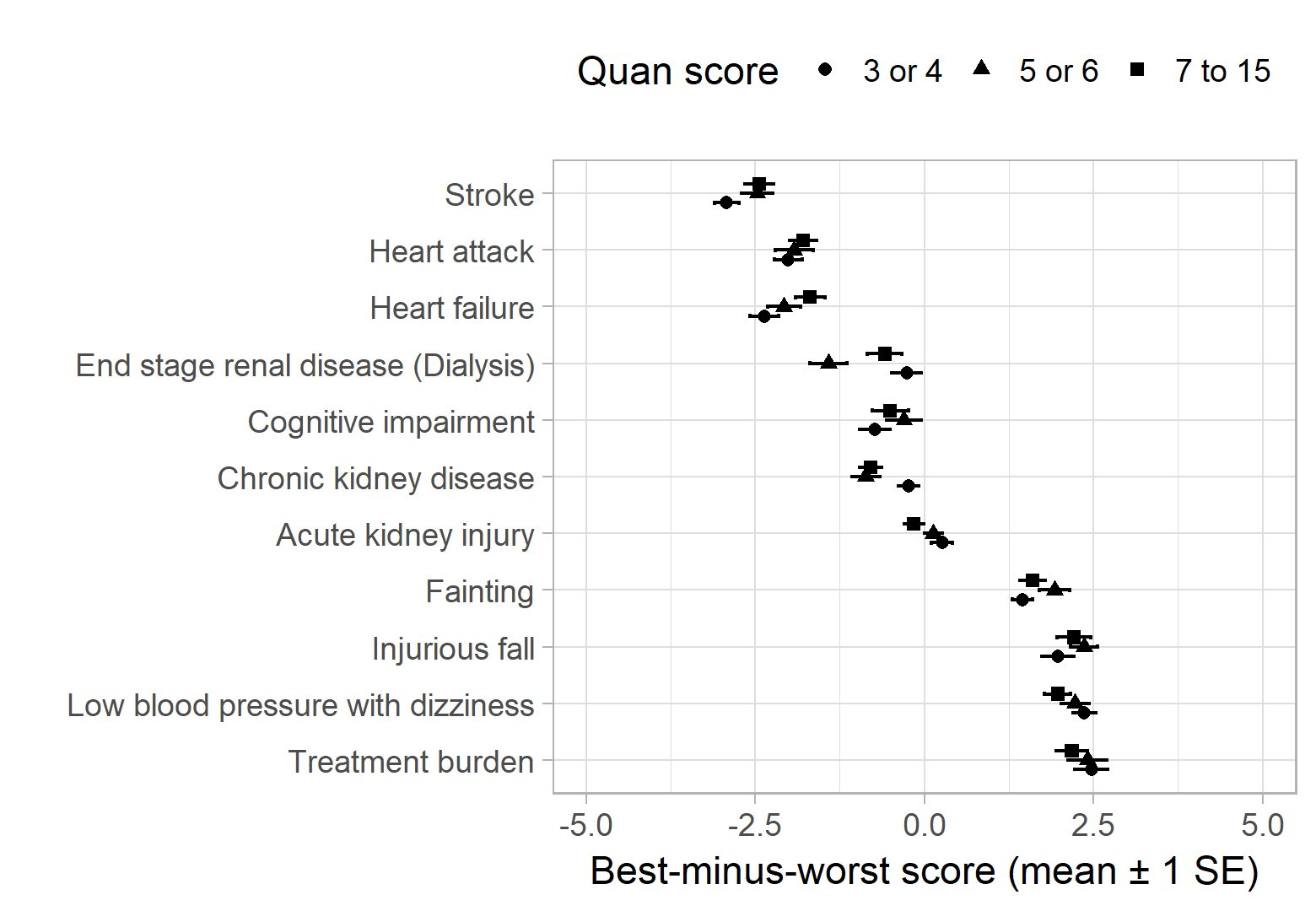


Figure S3: Subgroup analysis of best-minus-worst scores by Quan score.
The Quan adaptation of the Elixhauser comorbidity index is a score counting the number of chronic conditions a person has.


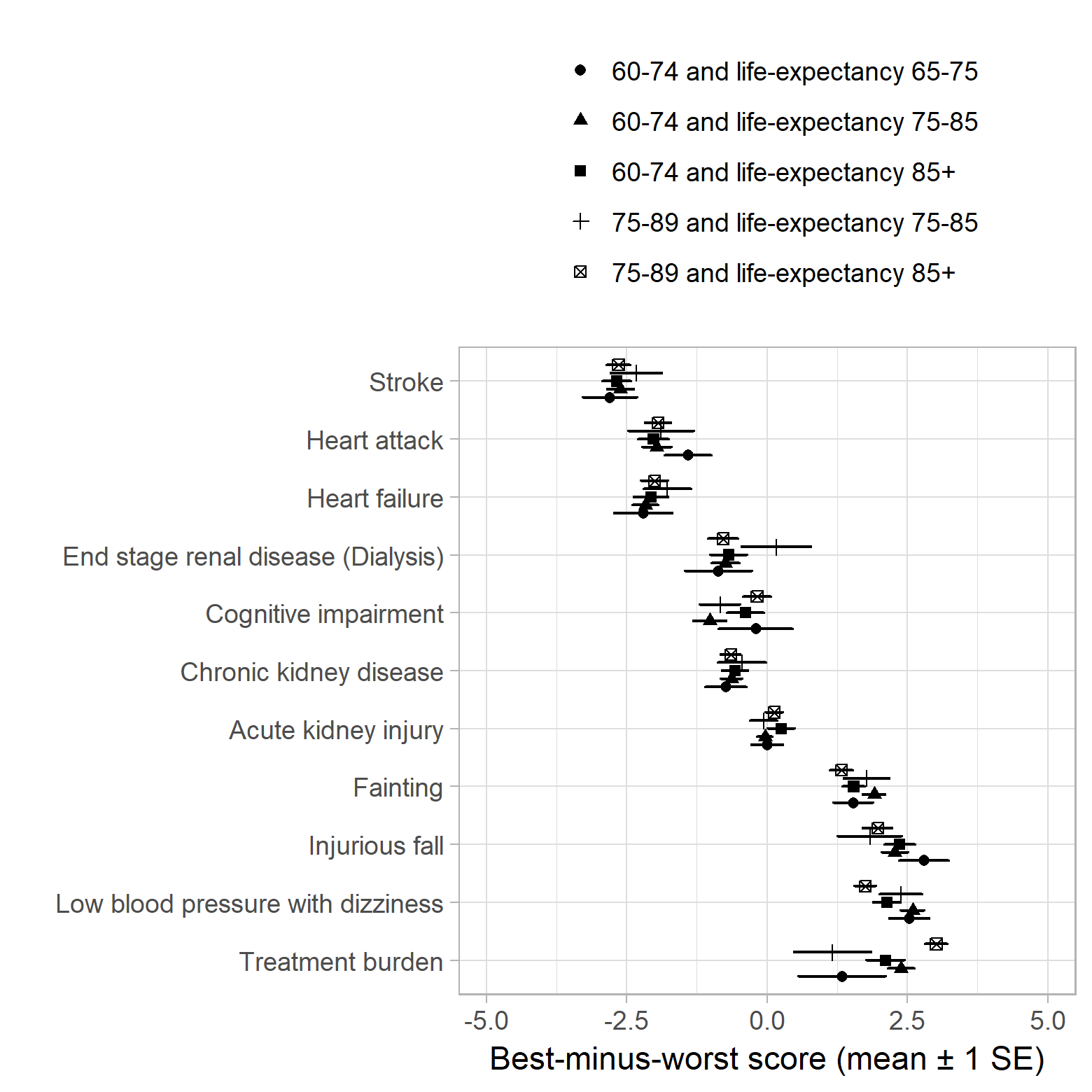


Figure S4: Subgroup analysis of best-minus-worst scores by self-reported life-expectancy, stratified by age.


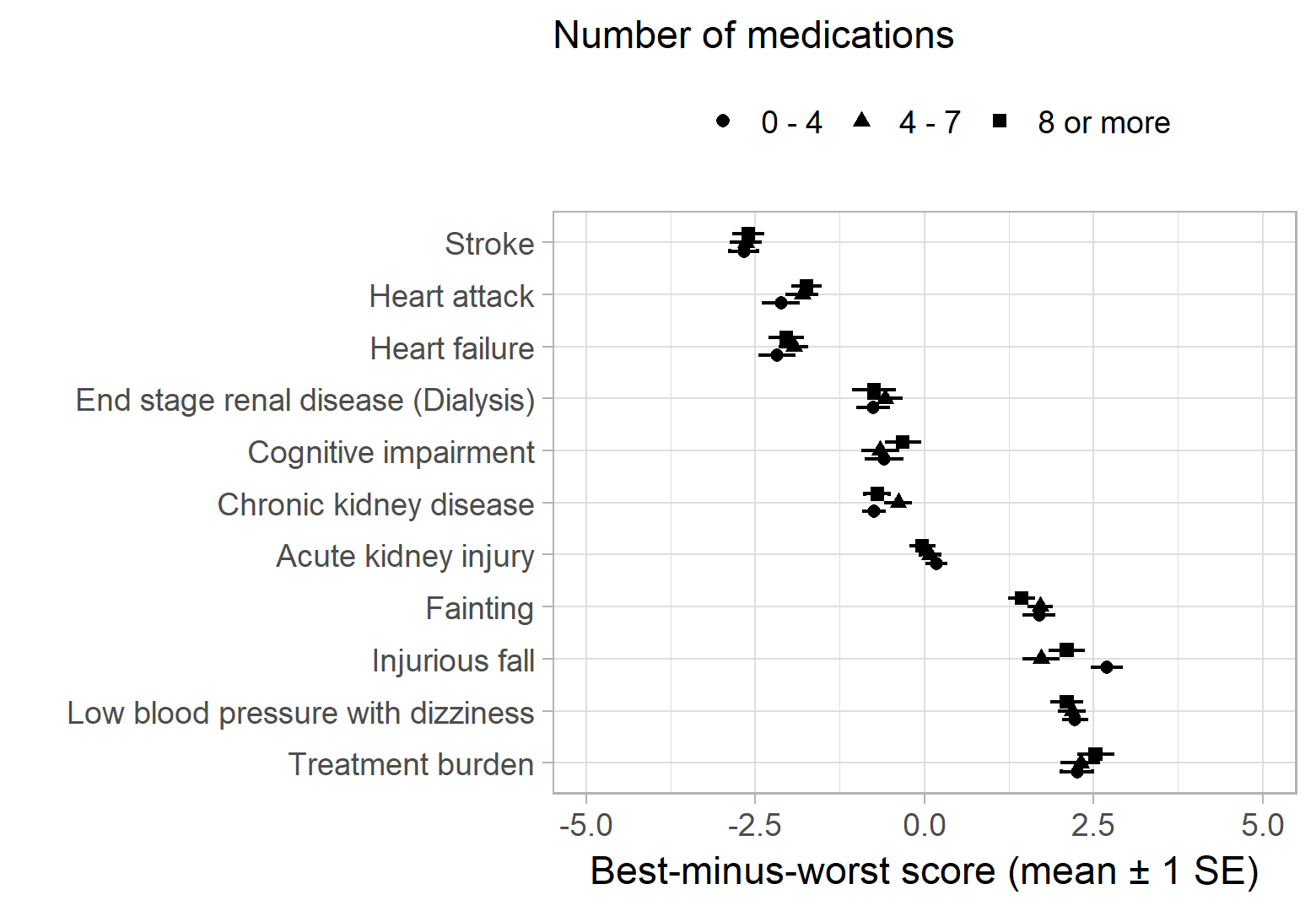


Figure S5: Subgroup analysis of best-minus-worst scores according to self-reported number of pills per day.


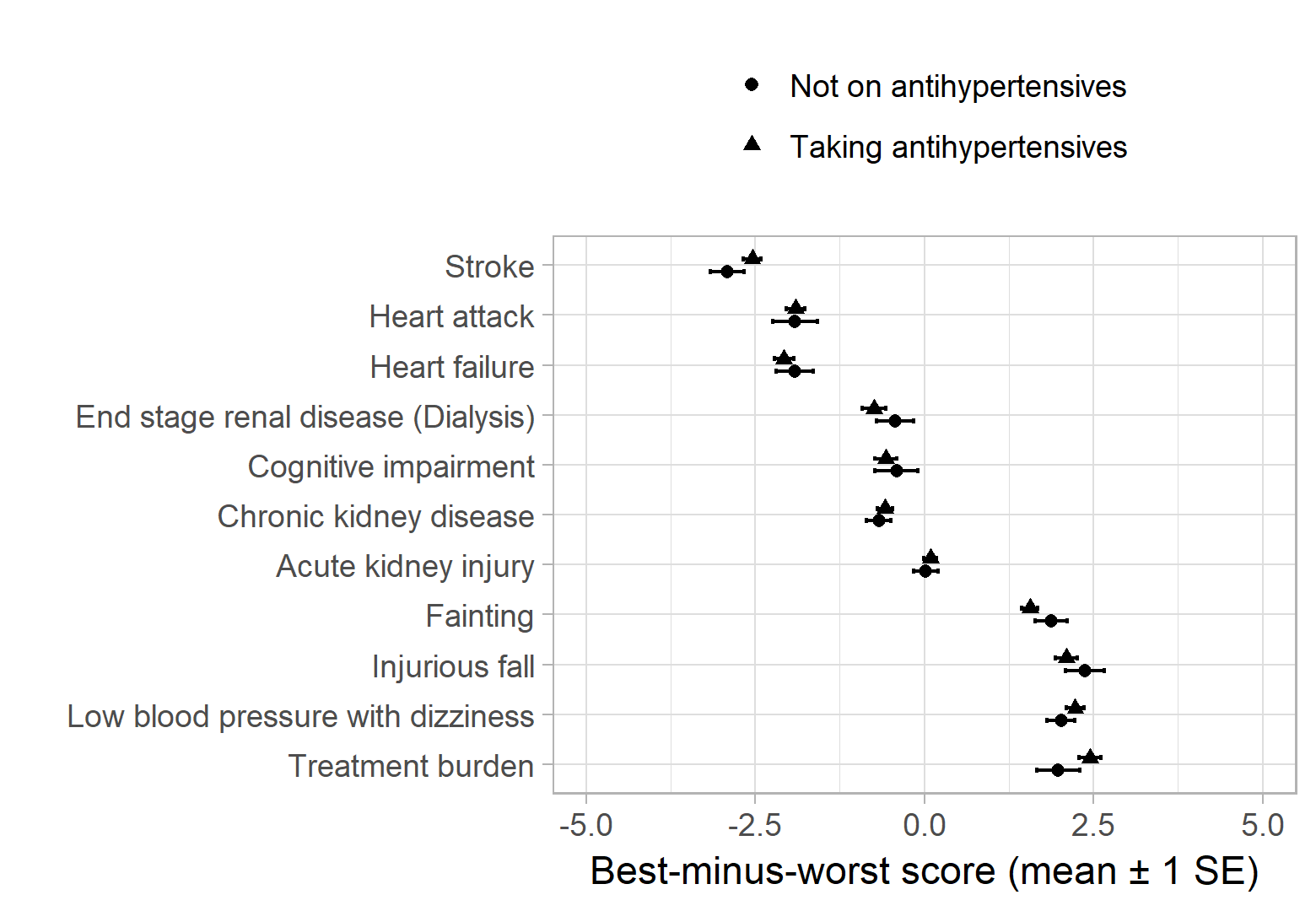


Figure S6: Subgroup analysis of best-minus-worst scores according to whether patients are on antihypertensive treatment or not.


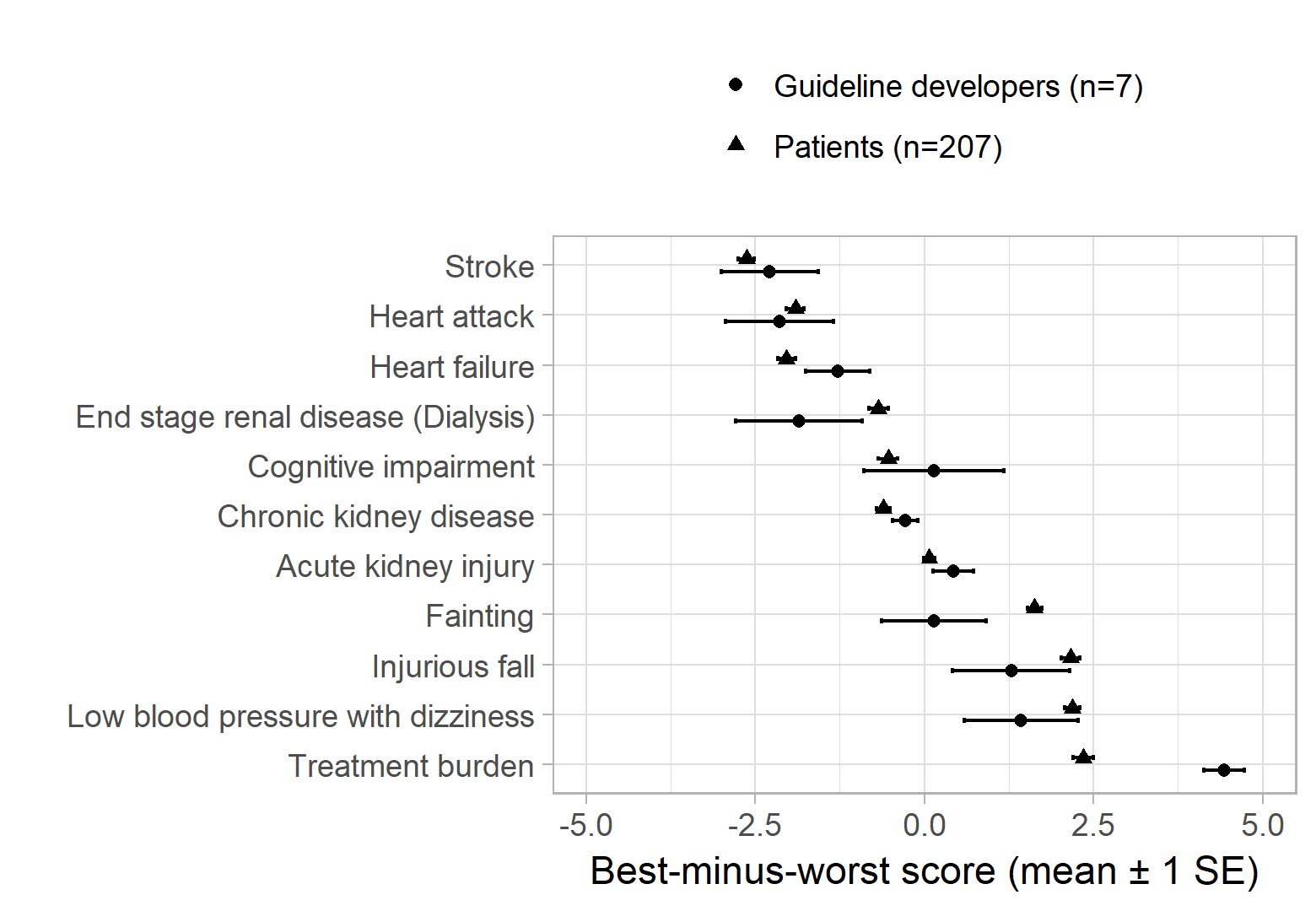


Figure S7: Best-minus-worst scores of patients compared to those of guideline developers.

Guideline developers worried less about treatment burden than patients did on average.
